# Comparative analysis of neutrophil-to-lymphocyte ratio and remnant cholesterol in predicting cardiovascular events and mortality in general adult population

**DOI:** 10.1038/s41598-023-49403-8

**Published:** 2023-12-15

**Authors:** Qian-Chen Wang, Zhen-Yu Wang

**Affiliations:** 1 Department of Cardiovascular Medicine, Xiangya Hospital, Central South University, Changsha, China; 2 Department of Cardiovascular Medicine, The Second Xiangya Hospital, Central South University, Renmin Road 139, Changsha, 410011 Hunan China

**Keywords:** Biomarkers, Cardiology

## Abstract

This study aimed to investigate the predictive value of neutrophil-to-lymphocyte ratio (NLR) and Remnant Cholesterol (Remnant-C) in relation to cardiovascular events and all-cause mortality in the general population. A population-based study. We conducted a retrospective cohort study analyzing data from the National Health and Nutrition Examination Survey (NHANES) spanning the years of 2011–2018, with follow-up for mortality status until December 31, 2019. Kaplan‒Meier and Cox proportional hazards regression analyses were used to evaluate the associations between NLR, Remnant-C, and cardiovascular events as well as all-cause mortality. Overall, 9409 individuals with both complete blood count and blood lipids were included in the analysis. Baseline NLR and Remnant-C were calculated. During the follow-up (median, 59.3 months), 177 cardiovascular events and 561 all-cause mortality occurred. In fully adjusted model, people with NLR > 2.26 were significantly associated with higher risk of cardiovascular events (HR 2.14, 95% CI 1.30–3.52, *P* < 0.001) and all-cause mortality (HR 1.66, 95% CI 1.30–2.12, *P* < 0.001). NLR exhibited a positive correlation with Remnant-C (r = 0.04, *P* < 0.001). Elevated NLR levels shown stronger association with cardiovascular events (HR 1.21, 95% CI 1.14–2.28, *P* < 0.001) compared with Remnant-C (HR 1.02, 95% CI 1.00–1.04, *P* = 0.020). Our findings suggest that NLR and Remnant-C are potential predictive markers for cardiovascular events in the general population. We observed a correlation between NLR and Remnant-C, and high NLR levels demonstrate a stronger association with the prediction of cardiovascular events and all-cause mortality compared with Remnant-C.

## Introduction

Cardiovascular disease (CVD) remains a significant global health challenge, and an estimated 32% of all global deaths are still due to coronary artery disease, stroke, and other vasculopathies^1,2^. Therefore, accurate risk assessment is crucial for prevention and management of CVD.

Chronical inflammation and lipid metabolism abnormalities have been recognized as significant contributors to cardiometabolic diseases development and progression respectively^3,4^.

Neutrophil-to-lymphocyte ratio (NLR), an easily obtainable biomarker, reflects the systemic inflammatory response^5,6^, and it has been shown to have prognostic value in various cardiovascular conditions, including coronary artery disease and stroke^7,8^.

Low-density lipoprotein cholesterol (LDL-C) has been extensively studied for its association with cardiovascular risk. However, recent research has highlighted the importance of Remnant cholesterol (Remnant-C), which represents the cholesterol content of triglyceride-rich lipoproteins, in independent prediction of cardiovascular events and outcomes^9–11^. Emerging evidence suggests that Remnant-C may play a distinct role in atherogenesis and cardiovascular risk beyond the traditional lipid profile^12–14^.

However, the relationship between Remnant-C and inflammation remains poorly understood. Moreover, no study has directly compared Remnant-C and the inflammatory marker NLR regarding the prediction of cardiovascular events in general population. Hence, the aim of this study is to investigate the relationship between NLR and Remnant-C and to compare the predictive abilities of NLR and Remnant-C in the context of cardiovascular events in general population by analyzing data from the National Health and Nutrition Examination Survey (NHANES).

## Methods

### Study design and participants

This study utilized data from the NHANES, a nationally representative survey managed by the National Center for Health Statistics (NCHS) of the Centers for Disease Control and Prevention (CDC). The NHANES collects health and nutrition data through interviews, physical examinations, and laboratory tests. The NHANES study required ethical approval of the NCHS Research Ethics Assessment Board. Participants, or their guardians, were fully informed about the study and provided signed informed consent. The NHANES study maintains strict participant privacy and confidentiality. The detailed NHANES data can be accessed by the public at https://www.cdc.gov/nchs/nhanes/.

The flowchart in Fig. S1 illustrates the participant selection process. The inclusion criteria were (1) aged over 18, (2) nonpregnant, (3) having complete blood count (CBC) with 5-part differential results, (4) having blood lipid profile results, and (5) eligible for mortality assessment. Individuals meeting the inclusion criteria from 2-years NHANES survey cycles between 2011 and 2018 were screened.

### Data collection and variables assessment

The collected data included demographics data (age, gender, education, and race), examination data (Body Mass Index, waist circumference, systolic and diastolic blood pressure), laboratory data (CBC, lipid profile, glycosylated hemoglobin), questionnaire data (smoke, alcohol use, diabetes, hypertension) and mortality data.

For laboratory test, blood samples were collected from participants after fasting for more than nine hours. The methods used to derive CBC parameters are based on the Beckman Coulter method of counting and sizing. The concentrations of triglyceride (TG), total cholesterol (TC) and high-density lipoprotein-cholesterol (HDL-C) were measured by Roche Modular P Chemistry Analyzer using enzymatic tests. The concentrations of LDL-C were calculated according to the Sampson formula^15^.

In this study, Remnant-C levels were calculated using the equation: Remnant-C(mg/dL) = TC-HDL-C-LDL-C. The NLR was determined by calculating the ratio between the neutrophil and lymphocyte counts obtained from peripheral blood samples.

### Outcomes

The de-identified and anonymized data from NHANES participants between 2011 and 2018 were linked to longitudinal Medicare and mortality data using the NHANES assigned sequence number. Mortality follow-up data were available from the date of survey participation until December 31, 2019. Cause-specific mortality, including heart diseases (I00-I09, I11, I13, I20-I51) and cerebrovascular disease (I60-I69), was assessed based on the 10th revision of the International Classification of Diseases (ICD-10). The main outcome variables of this study were cardiovascular events, including mortality from heart diseases and cerebrovascular diseases. All-cause mortality served as a secondary outcome.

### Statistical analysis

The NHANES-recommended weights were applied to the study analysis, allowing for representative population estimates. Continuous variables were analyzed using ANOVA and are reported as mean ± standard error of the mean (SEM). Categorical variables were assessed using chi-square tests and presented as percentages. Group differences were assessed using either the analysis of variance (ANOVA) or the Kruskal–Wallis H test for continuous variables, depending on the normality assumption. For categorical variables, the χ^2^ test was used to evaluate group differences.

The participants were classified into three NLR groups (low, middle, and high) based on the NLR tertiles within the entire cohort. Survival analysis was conducted using log-rank tests and Kaplan–Meier plots. Cox proportional hazards models, adjusted for confounders, including age, gender, smoking habit, body mass index (BMI), hypertension, diabetes mellitus (DM), levels of HbA1c and LDL-C, were utilized to estimate hazard ratios (HRs) with 95% confidence intervals (95%CIs) for cardiovascular events and all-cause mortality. Statistical significance was set at a two-sided* P*-value of 0.05. Data analyses were performed using SPSS 25.0 (IBM, Armonk, NY) and the R program (version 4.2.3; Vienna, Austria).

## Results

### Baseline characteristics according to tertiles of NLR

Overall, a total of 9409 individuals were included in the analysis, with a mean age of 49.47 years, comprising 48.8% men and 51.2% women. The baseline characteristics of the study participants are shown in Table 1 categorized into tertiles based on their NLR values. The participants were divided into three groups: low NLR tertile (NLR ≤ 1.57, tertile 1), middle NLR tertile (1.57 < NLR ≤ 2.26, tertile 2), and high NLR tertile (NLR > 2.26, tertile 3). Across the tertiles, there were differences in several baseline characteristics. There was a significant age difference between tertiles of NLR (P < 0.001). Additionally, in the high NLR tertile, there was a higher proportion of Non-Hispanic White (48.3%, *P* < 0.001), whereas the highest percentage of Non-Hispanic Black was in the low tertile of NLR (37.7%, *P* < 0.001). Individuals with high tertile NLR levels were more likely to be smokers, to have higher BMI and waist circumference. Regarding comorbidities, a higher prevalence of hypertension and DM was observed in the high NLR tertile. Furthermore, participants in the high NLR tertile had higher levels of HbA_1c_ and TG, while levels of high-density lipoprotein cholesterol were lower compared with other tertiles.

**Table 1:** Baseline characteristics by tertiles of NLR.

	Total N = 9409	Low n = 3183	Middle n = 3106	High n = 3120	*P* value
Age, years	49.47 ± 0.19	46.67 ± 0.31	48.15 ± 0.31	53.63 ± 0.33	< 0.001
Female, n (%)	4819 (51.2)	1659 (52.9)	1599 (51.5)	1561 (50.0)	0.237
Race					< 0.001
Mexican American	1284 (13.6)	409 (12.8)	477 (15.4)	398 (12.7)	
Other Hispanic	1009 (10.7)	316 (9.9)	357 (11.5)	336 (10.8)	
Non-Hispanic White	3547 (37.7)	871 (27.4)	1169 (37.6)	1507 (48.3)	
Non-Hispanic Black	2021 (21.5)	995 (31.3)	553 (17.8)	473 (15.2)	
Non-Hispanic Asian	1219 (13.0)	474 (14.9)	448 (14.4)	297 (9.5)	
Other race	329 (3.5)	118 (3.7)	102 (3.3)	109 (3.5)	
Education level, n (%)					0.093
Less than high school	2186 (23.2)	712 (22.4)	760 (24.5)	714 (22.9)	
High school or above	7213 (76.7)	2469 (77.6)	2341 (75.4)	2403 (77.0)	
Smoking, n (%)	3977 (42.3)	1193 (37.5)	1271 (40.9)	1513 (48.5)	< 0.001
Alcohol use, n (%)	6565 (69.8)	2180 (68.5)	2171 (69.9)	2214 (71.0)	0.097
Body mass index, kg/m^2^	28.86 ± 0.07	28.31 ± 0.11	28.76 ± 0.12	29.52 ± 0.13	< 0.001
Waist circumference, cm	99.13 ± 0.18	96.57 ± 0.29	98.89 ± 0.30	102.06 ± 0.33	< 0.001
Systolic blood pressure, mmHg	122.93 ± 0.19	121.83 ± 0.31	121.77 ± 0.31	125.26 ± 0.34	< 0.001
Diastolic blood pressure, mmHg	69.90 ± 0.12	70.28 ± 0.20	69.86 ± 0.21	69.56 ± 0.23	0.101
Hypertension, n (%)	3464 (36.8)	1019 (32.0)	1079 (34.7)	1366 (43.8)	< 0.001
Diabetes, n (%)	1286 (13.7)	316 (9.9)	376 (12.1)	594 (19.0)	< 0.001
HbA_1c_, %	5.56 ± 0.01	5.54 ± 0.01	5.54 ± 0.01	5.59 ± 0.01	0.005
Plasma concentration					
TG (mg/dl)	102.71 ± 0.54	98.57 ± 0.92	103.60 ± 0.93	106.06 ± 0.94	< 0.001
Total cholesterol (mg/dl)	187.56 ± 0.41	189.44 ± 0.71	190.01 ± 0.71	183.21 ± 0.71	< 0.001
LDL-C (mg/dl)	116.01 ± 0.40	117.47 ± 0.70	118.60 ± 0.70	111.93 ± 0.69	< 0.001
HDL-C (mg/dl)	53.70 ± 0.15	54.74 ± 0.26	53.45 ± 0.26	52.89 ± 0.26	< 0.001
Remnant-C (mg/dL)	18.03 ± 0.10	17.43 ± 0.16	17.99 ± 0.17	18.69 ± 0.16	< 0.001
WBC count (1000 cells/µL)	6.66 ± 0.02	5.90 ± 0.03	6.64 ± 0.03	7.45 ± 0.03	< 0.001
Lymphocyte count (1000 cells/µL)	2.01 ± 0.01	2.36 ± 0.01	2.03 ± 0.01	1.65 ± 0.01	< 0.001
Neutrophils count (1000 cell/µL)	3.87 ± 0.01	2.81 ± 0.02	3.84 ± 0.02	5.00 ± 0.03	< 0.001
NLR	2.09 ± 0.01	1.21 ± 0.00	1.90 ± 0.00	3.19 ± 0.02	< 0.001

Data are presented as mean ± SEM or as n (%). The tertile ranges were low (≤ 1.57), middle (1.57–2.26), and high (> 2.26). The *p*-value for the test of the difference across tertiles of NLR was obtained using the χ^2^ test for categorical variables, ANOVA for continuous variables, or the Kruskal–Wallis test for nonparametric comparisons.

### NLR levels and outcomes

The association between NLR levels and outcomes, including cardiovascular events and all-cause mortality, was also assessed. During the follow-up period of 59.3 months, 561 total deaths were recorded, including 177 cardiovascular events. The distributions of overall and cause-specific mortality across tertiles of NLR are shown in Table 2. The risk of both cardiovascular events and all-cause mortality were increased with increasing tertiles of NLR.

**Table 2:** The distributions of all-cause mortality and cardiovascular event across NLR tertiles.

	Total N = 9409	Low n = 3183	Middle n = 3106	High n = 3120	*p* value
All-cause mortality, n (%)	561 (6.0)	109 (3.4)	123 (4.0)	329 (10.5)	< 0.001
Cardiovascular event, n (%)	177 (1.9)	25 (0.8)	37 (1.2)	115 (3.7)	< 0.001

**Figure 1:**
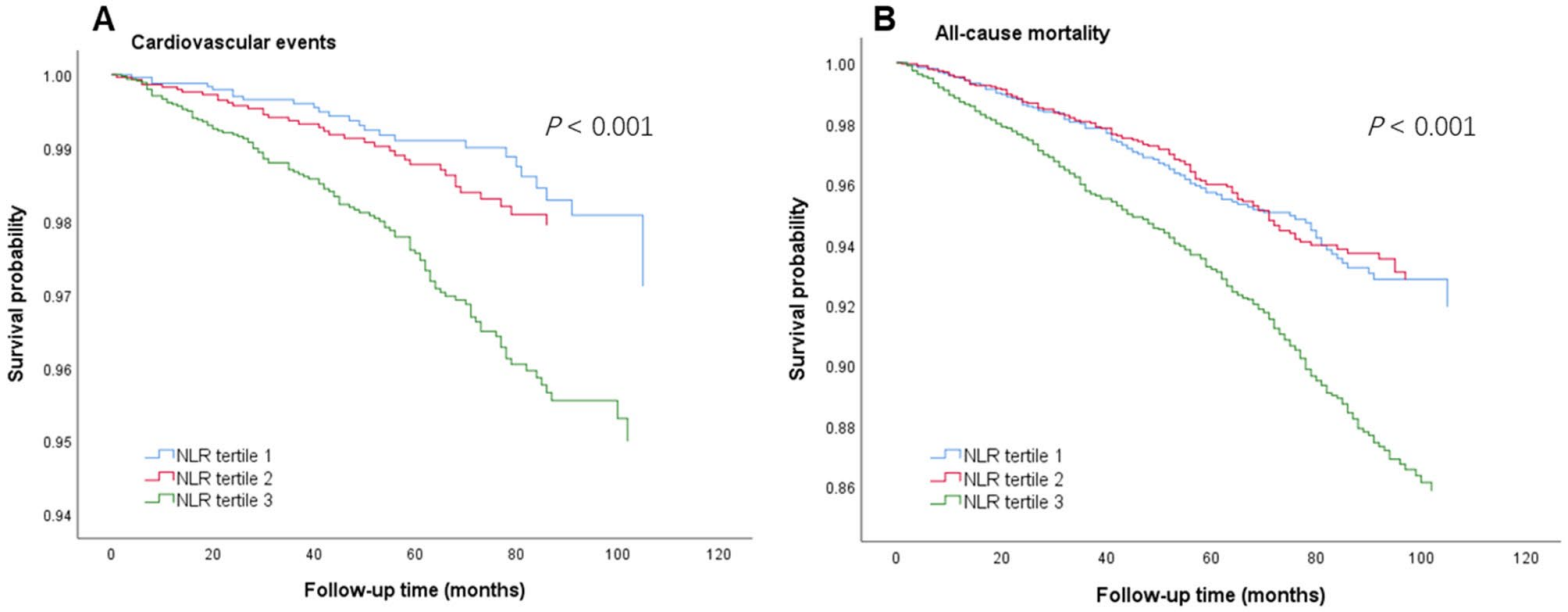
Kaplan‒Meier curve analysis for cardiovascular events and all-cause mortality according to the NLR tertiles.

However, in tertile analyses, significant difference was only observed between tertile 1 and 3 when analyzing the association of NLR levels with cardiovascular events (HR 2.14, 95% CI 1.30–3.52,* P* < 0.001) and all-cause mortality (HR 1.66, 95% CI 1.30–2.12, *P* < 0.001) (Table 3). In different models, after adjustment for confounding factors, there was still a significant positive association between tertile 3 and outcomes, as also shown in Fig. 1, further indicating that the baseline NLR level could be used as a prognostic marker for cardiovascular events and all-cause mortality in general adult population.

**Table 3:** Risk of all-cause mortality and cardiovascular events according to NLR level.

NLR group	Unadjusted HR (95% CI)	*P* value	Model 1 HR (95% CI)	*P* value	Model 2 HR (95% CI)	*P* value	Model 3 HR (95% CI)	*P* value
Cardiovascular events								
Tertile 1	REF		REF		REF		REF	
Tertile 2	1.50 (0.90–2.50)	0.116	1.26 (0.75–2.12)	0.378	1.21 (0.72–2.04)	0.464	1.11 (0.62–1.99)	0.718
Tertile 3	4.83 (3.135–7.447)	< 0.001	2.25 (1.43–3.54)	< 0.001	2.14 (1.36–3.38)	< 0.001	2.14 (1.30–3.52)	< 0.001
All-cause mortality								
Tertile 1	REF		REF		REF		REF	
Tertile 2	1.15 (0.89–1.48)	0.302	1.02 (0.78–1.33)	0.876	0.99 (0.76–1.29)	0.99	1.00 (0.75–1.32)	0.972
Tertile 3	3.174 (2.56–3.94)	< 0.001	1.79 (1.42–2.25)	< 0.001	1.74 (1.38–2.19)	< 0.001	1.66 (1.30–2.12)	< 0.001

Model 1: includes adjustments for age, gender, smoking habit, and BMI.

Model 2: in addition to the covariates from Model 1, Model 2 includes adjustments for hypertension diagnosis and diabetes diagnosis.

Model 3: in addition to the covariates from Model 2, Model 3 includes adjustments for levels of HbA1c, LDL-C and Remnant-C.

### Associations of NLR and blood lipids

Certain lipid molecules in the blood can also trigger inflammatory responses. Patients with higher expression levels in lipids profiles are at a significantly higher risk of CVD^16–18^. Therefore, we compared the relationship between NLR and TC, HDL-C, calculated LDL-C levels, and Remnant-C as shown in Table 4. Surprisingly, we found that NLR demonstrated a mild negative correlation with TC (r = -0.08,* P* < 0.001), LDL-C (r = − 0.08, *P* < 0.001), and HDL-C (r = − 0.04, *P* < 0.001). However, the NLR exhibited a positive correlation with Remnant-C (r = 0.04, *P* < 0.001), which presents in TG-rich lipoproteins. Previous research has shown that Remnant-C independently predicts cardiovascular events, regardless of LDL-C levels. These findings suggest that Remnant-C, rather than TC or LCL-C, may have a positive association with the inflammatory marker NLR.

**Table 4:** Pearson correlation coefficients r (and age- adjusted r) between blood lipids and NLR.

	NLR	Total cholesterol	LDL-C	HDL-C	Remnant-C
NLR	1				
Total cholesterol	− 0.08** (− 0.10)	1			
LDL-C	− 0.08** (− 0.09)	0.92** (0.93)	1		
HDL-C	− 0.04** (− 0.06)	0.21** (0.21)	− 0.09** (− 0.09)	1	
Remnant-C	0.04** (0.02)	0.18** (0.17)	0.05** (0.07)	− 0.32** (− 0.34)	1

**P < 0.01.

### Comparison of NLR and Remnant-C in predicting cardiovascular events and all-cause mortality

**Figure 2:**
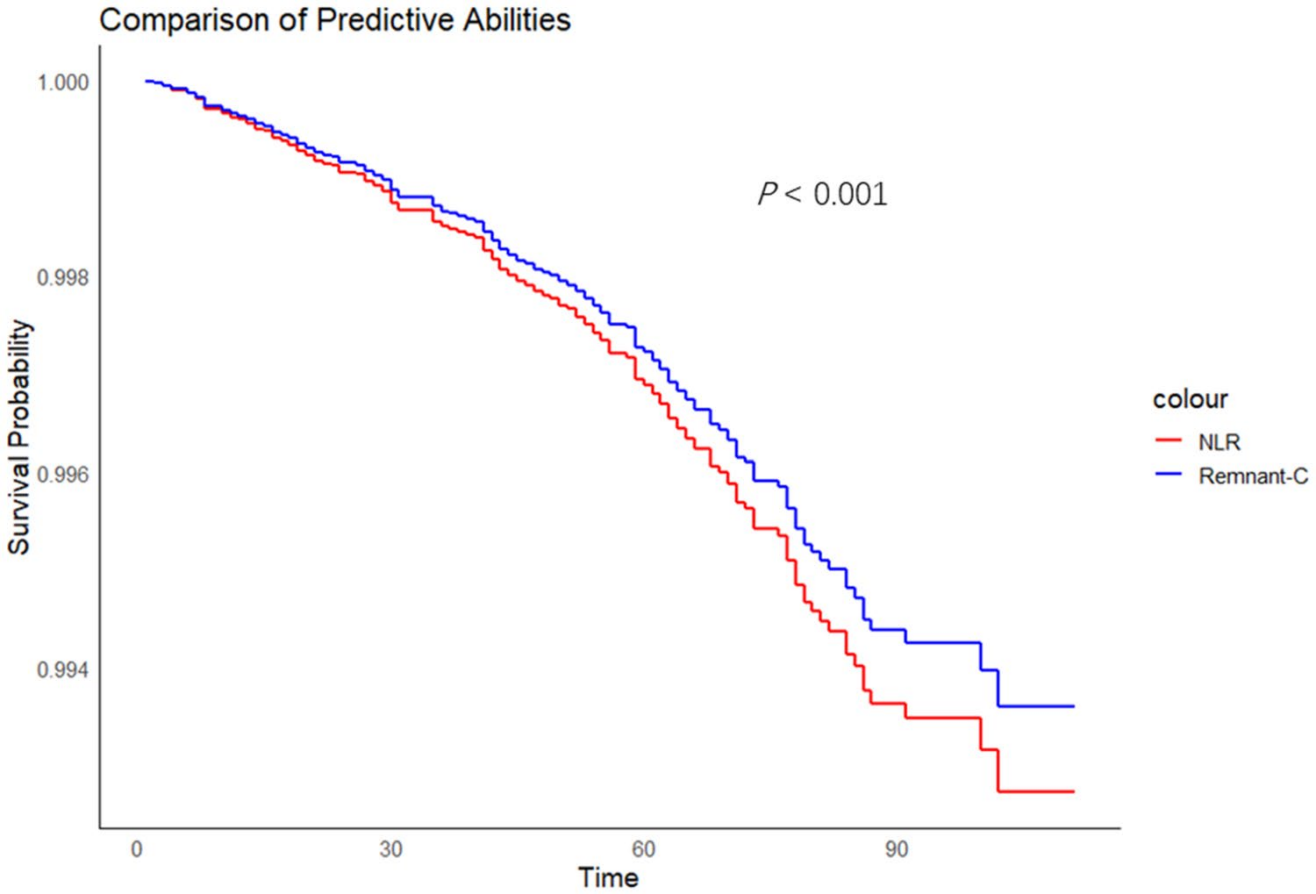
Adjusted association of Remnant-C and NLR with CVD events. Adjusted: Age, gender, BMI, hypertension, diabetes, and smoking.

**Figure 3:**
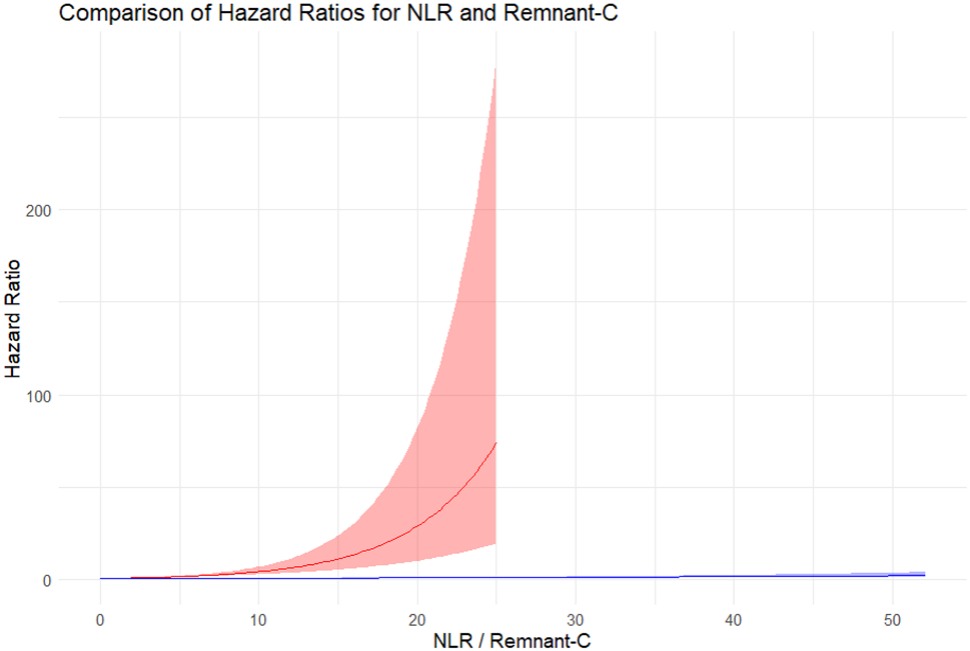
Comparing hazard ratios for NLR and Remnant-C in predicting cardiovascular events. Adjusted: Age, gender, BMI, hypertension, diabetes, and smoking. Red line and reddish area represent HR and 95% CI respectively.

Our study examined the predictive power of LDL-C and Remnant-C for cardiovascular events. We found that Remnant-C, not LDL-C, was linked to cardiovascular events (as shown in Supplementary Table 1), consistent with the findings of Castañer O et al.^11^. Given the mild positive correlation between NLR and Remnant-C, as well as our analysis showing the association between high NLR levels and cardiovascular events and all-cause mortality, we proceeded to compare the associations of NLR and Remnant-C with cardiovascular events and all-cause mortality using Cox proportional hazards models. The results are presented in Table 5. Interestingly, we found that both NLR (HR 1.21, 95% CI 1.14–1.28,* P* < 0.001) and Remnant-C (HR 1.02, 95% CI 1.00–1.04,* P* = 0.020) could predict cardiovascular events after adjusting for traditional cardiovascular risk factors. NLR exhibited a higher hazard ratio for predicting cardiovascular events, indicating a stronger association. Additionally, after adjusting for traditional cardiovascular risk factors, only NLR (HR 1.20, 95% CI 1.16–1.25,* P* < 0.001) remained significantly associated with all-cause mortality. Kaplan–Meier estimates (Fig. 2) and hazard ratio curves (Fig. 3) further highlight the differences between NLR and residual cholesterol in predicting cardiovascular events.

**Table 5:** Comparison of NLR and Remnant-C in prediction of cardiovascular events and all-cause mortality.

	HR	*p* value	Adjusted HR	*p* value
Cardiovascular events				
NLR	1.38 (1.33–1.44)	< 0.001	1.21 (1.14–1.28)	< 0.001
Remnant-C	1.03 (1.01–1.04)	0.001	1.02 (1.00–1.04)	0.020
All-cause mortality				
NLR	1.35 (1.32–1.39)	< 0.001	1.20 (1.16–1.25)	< 0.001
Remnant-C	1.02 (1.01–1.02)	0.001	1.01 (1.00–1.02)	0.294

Adjusted: Age, gender, BMI, hypertension, diabetes, and smoking.

## Discussion

In this retrospective analysis, we found that baseline NLR levels were significantly associated with cardiovascular events and all-cause mortality. The association between NLR and adverse cardiovascular outcomes highlights the potential role of inflammation in the development and progression of cardiovascular disease. The systemic inflammatory response reflected by the NLR may serve as a useful biomarker for identifying individuals at higher risk of cardiovascular events and death.

NLR reflects the balance between neutrophil-mediated inflammation and lymphocyte-mediated immune response^19–21^. Elevated NLR levels have been shown to be associated with a variety of pathological conditions, including atherosclerosis, plaque instability, and vascular dysfunction^22,23^. Martínez-Urbistondo et al.^24^, investigated the NLR as a marker of systemic endothelial dysfunction in asymptomatic subjects. Suárez-Cuenca et al.^25^, found that NLR was related to atherogenic progression.

On the other hand, recent studies have highlighted the importance of remnant cholesterol (Remnant-C), a relatively new concept representing the cholesterol content of triglyceride-rich lipoproteins, such as remnant chylomicrons, very low-density lipoprotein cholesterol (VLDL-C), and medium-density lipoproteins^26–29^. In the PREDIMED trial population, Remnant-C > 30 mg/dL was found to be the leading risk factor for the incidence of major cardiovascular event in patients at high cardiovascular risk^11^. Another study found that elevated Remnant-C is very common in patients admitted for acute coronary syndrome and Remnant-C level is associated with a higher risk of long-term death^30^. Remnant cholesterol may be a better indicator of atherosclerotic cardiovascular disease risk than LDL-C^31^.

To answer the question of whether Remnant-C correlates with the level of systemic inflammation, by analyzing the large-scale dataset from NHANES, we did observe a correlation between NLR and Remnant-C levels. This suggests a potential interplay between inflammation and lipid metabolism in cardiovascular risk. In light of these findings, we sought to elucidate the comparative predictive abilities for cardiovascular events of NLR and Remnant-C in general population. Notably, our results revealed a significant association between high NLR levels and increased risk of cardiovascular events, highlighting its strong prognostic value. Strikingly, when comparing NLR with Remnant-C, we found that NLR demonstrated superior predictive power, even after adjusting for traditional cardiovascular risk factors. These findings underscore the crucial role of inflammation in cardiovascular risk assessment and suggest that NLR may serve as a more robust predictor for adverse cardiovascular outcomes compared with Remnant-C.

It is worth noting that our study has several strengths. We utilized a large, nationally representative dataset, allowing for generalizability of the findings to the general population. Additionally, we employed rigorous statistical analyses and adjusted for confounding factors to enhance the robustness of our results.

However, several limitations should also be considered. First, our study relied on observational data, and therefore, causal relationships cannot be inferred. Second, the follow-up duration may not capture long-term outcomes beyond the available follow-up period. Third, although we adjusted for various confounding factors, residual confounding or unmeasured variables may still exist.

In conclusion, our study provides valuable insights into the relationship between NLR, Remnant-C, and cardiovascular outcomes. By demonstrating the superior predictive ability of NLR to Remnant-C, we underscore the importance of incorporating inflammation markers in cardiovascular risk assessment. Our findings emphasize the potential clinical utility of NLR as a risk stratification tool and highlight the need for targeted interventions aimed at reducing inflammation to improve cardiovascular outcomes. Further research is necessary to deepen our understanding of the complex interplay between inflammation, lipid metabolism, and cardiovascular disease, and to explore the therapeutic implications of targeting inflammation in the management of cardiovascular risk.

### Supplementary Information

Supplementary Information.

## Data Availability

All data used are publicly available from: URL: https://wwwn.cdc.gov/nchs/nhanes/Default.aspx and https://www.cdc.gov/nchs/data-linkage/mortality-public.htm.
