# Supplementary material for: Comparative analysis of neutrophil-to-lymphocyte ratio and remnant cholesterol in predicting cardiovascular events and mortality in general adult population
Source: Sci Rep. 2023 Dec 15;13:22362. doi: 10.1038/s41598-023-49403-8 (PMC10724289; doi:10.1038/s41598-023-49403-8)
Supplement: Supplementary file 1 — Supplementary Information. [file 41598_2023_49403_MOESM1_ESM.docx]

**Supplementary** **figure and table**


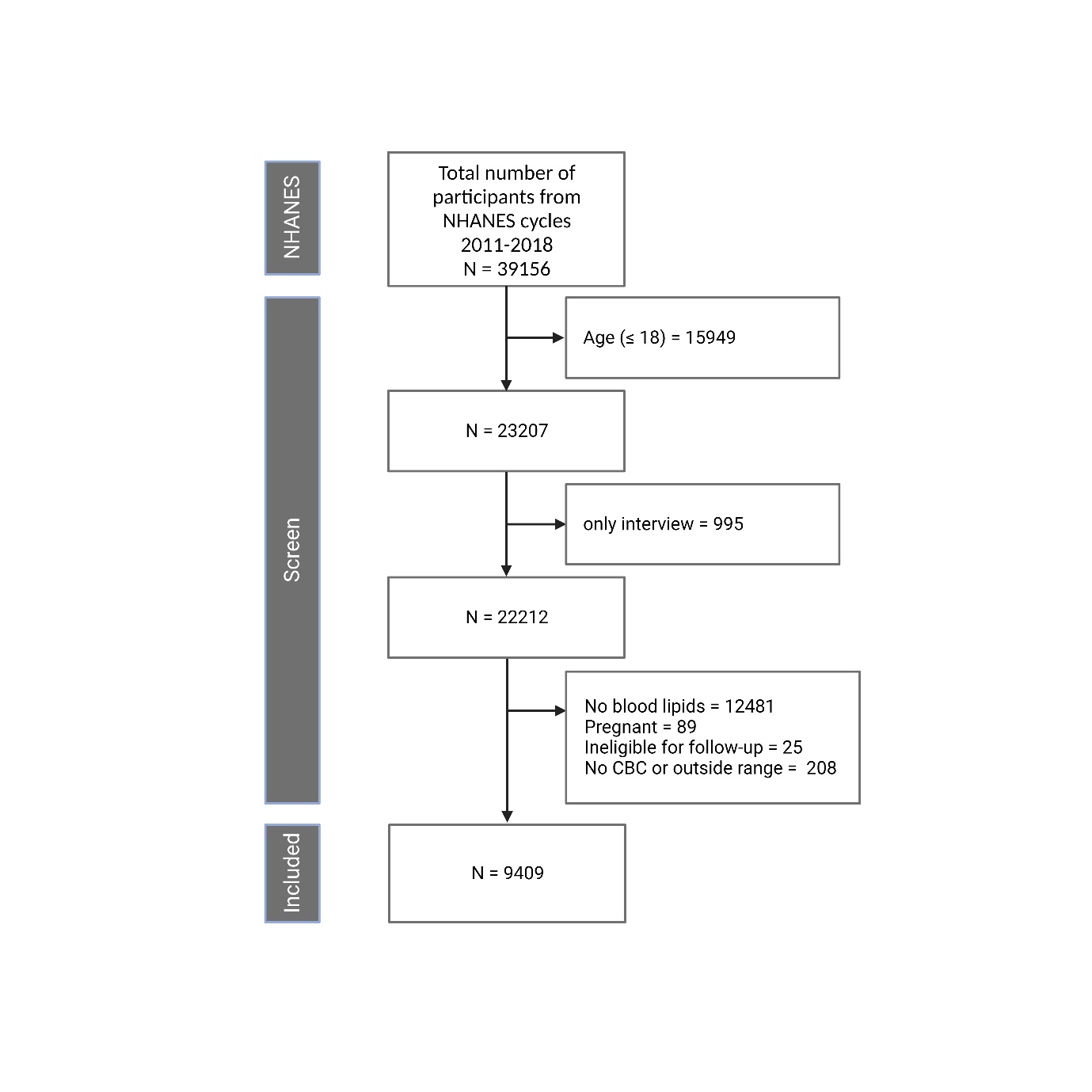


**Figure S1. Flow chart of the process for the selection of eligible participants.**

**Supplementary Table 1**

Comparison of LDL-C and Remnant-C in prediction of cardiovascular events

|  | HR | *p* value | Adjusted HR | *p* value |
| --- | --- | --- | --- | --- |
| Cardiovascular events |  |  |  |  |
| LDL-C | 0.713(0.595-0.855) | < 0.001 | 1.018(0.845-1.226) | 0.855 |
| Remnant-C | 1.03(1.01-1.04) | 0.001 | 1.02(1.00-1.04) | 0.020 |

Adjusted: Age, gender, BMI, hypertension, diabetes, and smoking.
